# The interplay between childhood trauma, cognitive biases, and cannabis use on the risk of psychosis in nonclinical young adults in Poland

**DOI:** 10.1192/j.eurpsy.2020.31

**Published:** 2020-03-23

**Authors:** Dorota Frydecka, Błażej Misiak, Kamila Kotowicz, Renata Pionke, Martyna Krężołek, Andrzej Cechnicki, Łukasz Gawęda

**Affiliations:** 1 Department of Psychiatry, Wroclaw Medical University, Wroclaw, Poland; 2 Department of Genetics, Wroclaw Medical University, Wroclaw, Poland; 3 Psychopathology and Early Intervention Lab, II Department of Psychiatry, The Medical University of Warsaw, Warsaw, Poland; 4 Department of Community Psychiatry, Chair of Psychiatry, Medical College Jagiellonian University, Krakow, Poland; 5 Experimental Psychopathology Lab, Institute of Psychology, Polish Academy of Sciences, Warsaw, Poland

**Keywords:** Childhood trauma, cognitive bias, psychotic-like experiences

## Abstract

**Background.:**

Childhood traumatic events are risk factors for psychotic-like experiences (PLEs). However, the mechanisms explaining how trauma may contribute to the development of PLEs are not fully understood. In our study, we investigated whether cannabis use and cognitive biases mediate the relationship between early trauma and PLEs.

**Methods.:**

A total sample of 6,772 young adults (age 26.6 ± 4.7, 2,181 male and 3,433 female) was recruited from the general population to participate in an online survey. We excluded 1,158 individuals due to a self-reported lifetime diagnosis of any mental disorder. The online survey included selected items from the following questionnaires: Traumatic Experience Checklist (TEC, 3 items), Childhood Experience of Care and Abuse Questionnaire (CECA.Q, 3 items), Cannabis Problems Questionnaire (CPQ, 10 items), Davos Assessment of Cognitive Biases Scale (DACOBS-18, 9 items), and Prodromal Questionnaire-16 (PQ-16). Mediation analyses were performed with respect to different categories of traumatic experiences (emotional, physical and sexual abuse as well as emotional neglect).

**Results.:**

Our results showed significant associations of any time of childhood trauma with higher scores of cannabis use (CPQ), cognitive biases (DACOBS), and PLEs (PQ-16) (*p* < 0.001). We found a direct effect of childhood trauma on PLEs as well as significant indirect effect mediated through cannabis use and cognitive biases. All models tested for the effects of specific childhood adversities revealed similar results. The percentage of variance in PQ-16 scores explained by serial mediation models varied between 32.8 and 34.2% depending on childhood trauma category.

**Conclusion.:**

Cannabis use and cognitive biases play an important mediating role in the relationship between childhood traumatic events and the development of PLEs in a nonclinical young adult population.

## Introduction

Psychotic-like experiences (PLEs) are defined as subclinical psychotic phenomena that include perceptual anomalies and delusion-like experiences in the absence of overt psychotic illness [1]. PLEs have been found to occur in 5–8% of nonclinical populations [2]. They have been associated with impairments in functioning [3], help-seeking behaviors [3,4], psychiatric diagnoses [3,4], self-harm thoughts and behaviors [5,6], as well as increased suicidality [3,7]. Moreover, PLEs have been linked to risk for developing a psychotic disorder and with many of the same risk factors as psychotic disorder, such as exposure to traumatic life events or cannabis use [2].

The association between early traumatic experiences and PLEs has been shown both in population-based studies [8], as well as among help-seeking adolescents and young adults [9]. It has been shown that even after controlling demographic factors and comorbid mental disorders, the relationship between traumatic life events and PLEs is fairly significant with odds ratios ranging from 3 to 11 as presented by a recent meta-analysis [10]. Moreover, prospective studies have shown that trauma exposure predates the onset of psychosis [11], and having a history of trauma is related to a more severe symptomatic manifestation, unfavorable course, and higher rates of treatment resistance [12].

Cannabis use has been increasing over the past decades and age at first use has been decreasing, which is of particular concern since the brain, which continues to develop in the adolescence, may be vulnerable to toxic effects of cannabis [13]. Early trauma [14] and later traumatic life events [15] have been associated with an increased likelihood of cannabis use. Independent contributions of childhood physical and sexual abuse to cannabis use were observed with no effect of witnessing parental violence [16]. Moreover, it has also been shown that childhood trauma predicts the transition from cannabis initiation to cannabis use disorder [17]. Interestingly, the effect of childhood maltreatment and cannabis abuse on pathogenesis of psychosis is neither fully confounded by other risk factors [10] nor can by explained by the gene–environment interactions [18].

Cannabis use has been repeatedly associated with the continuum of psychotic experiences, ranging from subthreshold psychotic symptoms to clinical high risk for psychosis. It has been demonstrated that PLEs are more prevalent among cannabis users in the general population when compared to nonusers [19–24]. Moreover, there is a dose–response relationship between the frequency of cannabis consumption and increased risk for psychosis [25,26]. Moreover, it has been shown using a longitudinal study design that the experience of childhood trauma moderates the association between cannabis and psychosis in a dose-dependent, extra-linear fashion. In two independent population-based samples, it has been found that severe maltreatment was associated with the greatest effect of cannabis in a later expression of psychosis [27]. This is in line with previous animal and human research showing that early life stress may result in an altered behavioral response to dopamine agonists later in life [28,29].

Despite numerous studies showing the association between early trauma and PLEs/psychosis, the mechanisms by which trauma influences the development of psychotic symptoms remain unclear. Several models focusing on psychological and biological mechanisms have been suggested so far and various mediation models have been proposed linking childhood and adolescent trauma with PLEs in nonclinical samples, including different variables such as: perceived stress, external locus of control, negative self-schemas, negative other-schemas [8], cognitive biases [30–32], resilience [32], dissociation [8,33], depressive symptoms [34], self-disturbances [30,31,35], insecure attachment styles [30], borderline personality features [9], and aberrant salience [36].

So far, the significance of cannabis use [13,27,37,38] as well as cognitive biases [30–32] in the relationship between early adversity and PLEs has been examined. However, to the best of our knowledge, to date, there are no studies addressing both risk factors simultaneously. Existing evidence suggests that linear models directly linking a history of childhood trauma, cannabis use and PLEs might be insufficient to understand causal mechanisms. Thus, in our study, we aimed at investigating serial mediation models including the interplay between cannabis use and cognitive biases to provide more detailed explanatory model for the relationship between exposure to early trauma and psychosis proneness.

## Methods

### Participants

A total sample of 6,772 young adults (age 26.6 ± 4.7; 2,181 male and 3,433 female) was enrolled from the general population to participate in an online survey using the Computer Assisted Web Interview (CAWI) method. Completing the online survey took on average around 20–30 min. We created the Research Consortium between medical universities in three big cities in Poland (Warsaw, Cracow, and Wroclaw) to investigate the relationship between early trauma, cognitive biases, and risk of psychosis. Participants were recruited from these cities with a range of 640,000–1,700,000 inhabitants. Exclusion criteria were as follows: history of substance dependence in the previous 6 months, history of psychotic or neurological disorders, and taking antipsychotic medication. The study was approved by ethics committee of the Medical University of Warsaw. Participants provided their informed consent to participate in the study. Demographic and clinical characteristics of our sample are shown in Table 1.

**Table 1.** General characteristics of the sample.

	Mean ± SD or *n* (%)
Age	26.6 ± 4.7
Sex	
Male	2,181 (38.8%)
Female	3,433 (61.2%)
Professional situation	
Study	1,924 (34.3%)
Work	4,126 (73.5%)
Unemployed	329 (5.9%)
Rent	26 (0.5%)
Education	
Primary	154 (2.7%)
Secondary	1,837 (32.7%)
Vocational	196 (3.5%)
Incomplete higher	884 (15.7%)
Higher	2,543 (45.3%)
Type of childhood adversity	
Emotional abuse	2,060 (36.7%)
Emotional neglect	2,558 (45.6%)
Physical abuse	2,880 (51.3%)
Sexual abuse	757 (13.5%)
Any childhood adversities	3,919 (69.8%)
Questionnaires	
CPQ	2.6 ± 2.3
PQ-16	8.4 ± 5.9
DACOBS	26.0 ± 8.5

Abbreviations: CPQ, Cannabis Problems Questionnaire; DACOBS, Davos Assessment of Cognitive Biases Scale; PQ-16, Prodromal Questionnaire.

### Measures

#### Childhood trauma

Early exposure to trauma was assessed using selected items from the Traumatic Events Checklist (TEC) [39] and from the Childhood Experience of Care and Abuse Questionnaire (CECA.Q) [40]. We assessed emotional abuse, emotional neglect, and bullying with three items from the TEC, while sexual harassment and sexual abuse were assessed by three items from the CECA.Q. A detailed description of selected items was provided elsewhere [5]. Sample questions from the TEC are: “When you were a child or a teenager, have you ever felt emotionally neglected (e.g., being left alone, insufficient affection) by your parents, brothers, or sisters?” or “When you were a child or a teenager, have you ever felt emotionally abused (e.g., being belittled, teased, called names, threatened verbally, or unjustly punished) by your parents, brothers, or sisters?” Sample questions from the CECA.Q are: “When you were a child or a teenager, did you have any unwanted sexual experiences?” or “Can you think of any upsetting sexual experiences before age 17 with a related adult or someone in authority, for example, teacher?” We used the Polish version of the items that were prepared using the back-translation procedure. In our sample, the Cronbach’s alpha for selected items was 0.66.

#### Cognitive biases

Cognitive biases were measured with the Davos Assessment of Cognitive Biases Scale (DACOBS-18) [41]. We included two subscales that assess attention to threat biases (items 6, 7, 24, and 29) and safety behaviors—behavioral coping strategies (items 27, 31, 33, 34, and 35). These subscales have been proven to be best predictors of psychosis risk (for more detailed information about the chosen items see [5]). Sample questions from the DACOBS-18 are: “People cannot be trusted,” “Things went wrong in my life because of other people,” “People make my life miserable,” “People treat me badly for no reason,” “People I do not know are dangerous,” or “I do not go out after dark,” “I do not answer phone calls, to be on the safe side,” “I always sit near the exit to be safe,” “I do not answer phone calls, to be on the safe side,” “There is usually only one explanation for a single event.” We used the Polish version of the DACOBS-18 [42]. In our sample, the Cronbach alpha was 0.81.

#### Psychotic-like experiences (PLEs)

The Prodromal Questionnaire-16 (PQ-16) [43] was used to screen for the risk of psychosis operationalized as a presence of PLEs. It is a 16-item self-report questionnaire that consists of 9 items of the perceptual abnormalities/hallucinations subscale, 5 items referring to unusual thought content/delusional ideas/paranoia, and 2 negative symptoms. In our study, we used only the items related to the attenuated positive symptoms and we excluded two items associated with depression and anxiety symptoms. Sample questions from PQ-16 are: “I felt as if I had no control over my own ideas and/or thoughts.” In our study, the Polish version of PQ-16 was used that was prepared using a back-translation procedure and was used previously [30]. The Cronbach’s alpha in our sample was 0.87.

#### Cannabis use

Cannabis use was assessed using the CPQ [44]. We included the following 10 questions out of 16 questions from the CPQ referring to experiences with cannabis use in the preceding 12 months: (a) “Have you tended to smoke more on your own than you used to?”; (b) “Have you been neglecting yourself physically?”; (c) “Have you felt depressed for more than a week?”; (d) “Have you been so depressed you felt like doing away with yourself?”; (e) “Have you given up recreational activities you once enjoyed for smoking?”; (f) “Do you find it hard to get the same enjoyment from your usual interests?”; (g) “Have you felt more antisocial after smoking?”; (h) “Have you been concerned about a lack of motivation?”; (i) “Have you worried about feelings of personal isolation or detachment?”; and (j) “Do you usually have a smoke in the morning, to get yourself going?” The Cronbach’s alpha in our sample was 0.77.

### Data analysis

**Figure 1.**
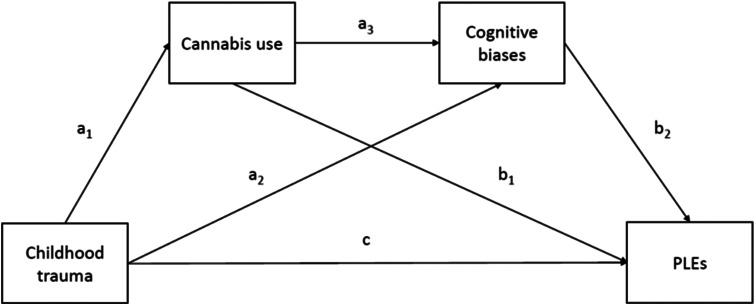
Model for serial mediation (direct and Indirect effects).

Before data analysis, given the fact that we were interested in nonclinical PLEs, individuals with a self-reported lifetime diagnosis of any mental disorders were excluded. Distribution of the PQ-16, DACOBS, and CPQ scores was non-normal according to the Kolmogorov–Smirnov test. Therefore, correlations between these variables were assessed using the Spearman rank correlation coefficients. Similarly, bivariate comparisons of these scores between individuals with and without any type or specific childhood adversities were performed using the Mann–Whitney *U* test. The alpha criterion level was set at 0.05 in bivariate analyses. The PROCESS macro was used to perform serial mediation analysis (Model 6) [45]. The bootstrap estimation with 5,000 samples was applied to test indirect effects. The model tested in our study was presented in Figure 1. The history of specific childhood adversities and history of any childhood traumatic events was implemented as an independent variable, while the PQ-16 score was used as a dependent variable. The DACOBS score and the CPQ score were implemented as mediators. Age, sex, and education level were added as covariates. Mediation was considered significant if the 95% confidence intervals (CI) did not include zero [46]. Statistical analysis was performed using the Statistical Package for Social Sciences, version 20 (SPSS Inc., Chicago, IL).

## Results

Out of 6,772 participants, we excluded 1,158 individuals (17.1%) due to a self-reported lifetime diagnosis of any mental disorders. We analyzed data from all individuals who completed the online survey. We included patients with and without traumatic life events and we did not apply any threshold criteria for any questionnaires used in the study. General characteristics of the sample were provided in Table 1. The majority of participants were females, had higher education and were employed on the day of assessment.

The following correlations appeared to be significant (*p* < 0.001): (a) between the CPQ and DACOBS scores (*r* = 0.375); (b) between the CPQ and PQ-16 scores (*r* = 0.465); and (c) between the PQ-16 and the DACOBS scores (*r* = 0.435). Similarly, a history of all specific childhood adversities and any type of childhood trauma were associated with higher scores of CPQ, DACOBS, and PQ-16 (Table 2).

**Table 2.** The level of cannabis use, cognitive biases and psychotic-like experiences with respect to a history of childhood adversities.

	Trauma(+)	Trauma(−)	*p*
Emotional abuse	*n* = 2,060	*n* = 3,554	
CPQ	3.2 ± 2.3	2.2 ± 2.1	<0.001
DACOBS	28.3 ± 8.45	24.6 ± 8.2	<0.001
PQ-16	10.8 ± 6.2	7.1 ± 5.3	<0.001
Emotional neglect	*n* = 2,558	*n* = 3,056	
CPQ	3.15 ± 2.3	2.0 ± 2.1	<0.001
DACOBS	27.9 ± 8.4	24.3 ± 8.2	<0.001
PQ-16	10.4 ± 5.9	6.8 ± 5.4	<0.001
Physical abuse	*n* = 2,880	*n* = 2,734	
CPQ	3.1 ± 2.3	2.0 ± 2.0	<0.001
DACOBS	27.4 ± 8.5	24.5 ± 8.3	<0.001
PQ-16	10.1 ± 6.7	6.7 ± 5.4	<0.001
Sexual abuse	*n* = 757	*n* = 4,857	
CPQ	3.2 ± 2.4	2.5 ± 2.2	0.001
DACOBS	29.7 ± 9.2	25.4 ± 8.2	<0.001
PQ-16	11.7 ± 6.8	7.9 ± 5.6	<0.001
Any type of trauma	*n* = 3,919	*n* = 1,695	
CPQ	2.9 ± 2.3	1.7 ± 1.9	<0.001
DACOBS	27.1 ± 8.3	23.5 ± 8.4	<0.001
PQ-16	9.8 ± 5.8	5.3 ± 5.0	<0.001

Abbreviations: CPQ, Cannabis Problems Questionnaire; DACOBS, Davos Assessment of Cognitive Biases Scale; PQ-16, Prodromal Questionnaire.

The results of serial mediation analysis were shown in Table 3 and Figure 1. All models testing for the effects of specific childhood adversities or a history of any childhood trauma revealed similar results and included age, sex, and education level as covariates.

**Table 3.** Serial mediation analysis to identify direct and indirect effects of childhood trauma on psychotic-like experiences.

Effect	Path	Coeff.	SE	95% CI	
				LLCI	ULCI
Emotional abuse					
Direct effect of CT on cannabis use	a_1_	0.9479***	0.1496	0.6543	1.2414
Direct effect of CT on cognitive biases	a_2_	2.4549***	0.5144	1.4454	3.4645
Direct effect of cannabis use on cognitive biases	a_3_	1.2220***	0.1135	0.9993	1.4448
Direct effect of cannabis use on PLEs	b_1_	0.7566***	0.0747	0.6100	0.9031
Direct effect of cognitive biases on PLEs	b_2_	0.2133***	0.0209	0.1723	0.2543
Direct effect of CT on PLEs	c	1.6438***	0.3221	1.0115	2.2760
Total indirect effect	ab	1.4879	0.2149	1.0776	1.9226
Indirect effect through cannabis use	a_1_b_1_	0.7171	0.1410	0.4546	1.0079
Indirect effect through cognitive biases	a_2_b_2_	0.5237	0.1264	0.2902	0.7826
Indirect effect through cannabis use and cognitive biases	a_1_a_3_b_2_	0.2471	0.0553	0.1490	0.3630
Emotional neglect					
Direct effect of CT on cannabis use	a_1_	1.1336***	0.1492	0.8408	1.4265
Direct effect of CT on cognitive biases	a_2_	2.2282***	0.5243	1.1993	3.2571
Direct effect of cannabis use on cognitive biases	a_3_	1.2140***	0.1149	0.9886	1.4395
Direct effect of cannabis use on PLEs	b_1_	0.7614***	0.0758	0.6126	0.9101
Direct effect of cognitive biases on PLEs	b_2_	0.2199***	0.0210	0.1787	0.2611
Direct effect of CT on PLEs	c	1.1437**	0.3292	0.4977	1.7898
Total indirect effect	ab	1.6558	0.2189	1.2525	2.0962
Indirect effect through cannabis use	a_1_b_1_	0.8631	0.1504	0.5901	1.1825
Indirect effect through cognitive biases	a_2_b_2_	0.4900	0.1276	0.2543	0.7553
Indirect effect through cannabis use and cognitive biases	a_1_a_3_b_2_	0.3027	0.0613	0.1933	0.4351
Physical abuse					
Direct effect of CT on cannabis use	a_1_	1.0117***	0.1508	0.7158	1.3076
Direct effect of CT on cognitive biases	a_2_	2.0724**	0.5233	1.0453	3.0995
Direct effect of cannabis use on cognitive biases	a_3_	1.2354***	0.1142	1.0112	1.4596
Direct effect of cannabis use on PLEs	b_1_	0.7567***	0.0752	0.6092	0.9043
Direct effect of cognitive biases on PLEs	b_2_	0.2181***	0.0209	0.1771	0.2591
Direct effect of CT on PLEs	c	1.4384***	0.3264	0.7978	2.0789
Total indirect effect	ab	1.4901	0.2029	1.1149	1.9039
Indirect effect through cannabis use	a_1_b_1_	0.7656	0.1415	0.5060	1.0593
Indirect effect through cognitive biases	a_2_b_2_	0.4520	0.1216	0.2274	0.7045
Indirect effect through cannabis use and cognitive biases	a_1_a_3_b_2_	0.2726	0.0573	0.1690	0.3962
Sexual abuse					
Direct effect of CT on cannabis use	a_1_	0.7357**	0.2032	0.3369	1.1344
Direct effect of CT on cognitive biases	a_2_	3.3727***	0.6773	2.0433	4.7021
Direct effect of cannabis use on cognitive biases	a_3_	1.2697***	0.1117	1.0487	1.4870
Direct effect of cannabis use on PLEs	b_1_	0.7992***	0.0748	0.6524	0.9460
Direct effect of cognitive biases on PLEs	b_2_	0.2208***	0.0211	0.1794	0.2623
Direct effect of CT on PLEs	c	1.1598*	0.4297	0.3165	2.0031
Total indirect effect	ab	1.5387	0.1861	0.4064	1.1234
Indirect effect through cannabis use	a_1_b_1_	0.5879	0.1740	0.2704	0.9497
Indirect effect through cognitive biases	a_2_b_2_	0.7448	0.1861	0.4064	1.1234
Indirect effect through cannabis use and cognitive biases	a_1_a_3_b_2_	0.2060	0.0649	0.0917	0.3431
Any childhood adversities					
Direct effect of CT on cannabis use	a_1_	1.2001***	0.1908	0.8256	1.5746
Direct effect of CT on cognitive biases	a_2_	2.4432**	0.6618	1.1442	3.7421
Direct effect of cannabis use on cognitive biases	a_3_	1.2828***	0.1143	1.0584	1.5072
Direct effect of cannabis use on PLEs	b_1_	0.7717***	0.0755	0.6235	0.9198
Direct effect of cognitive biases on PLEs	b_2_	0.2250***	0.0208	0.1841	0.2658
Direct effect of CT on PLEs	c	1.6244***	0.4119	0.8160	2.4328
Total indirect effect	ab	1.8221	0.2546	1.3317	2.3237
Indirect effect through cannabis use	a_1_b_1_	0.9261	0.1687	0.6262	1.2840
Indirect effect through cognitive biases	a_2_b_2_	0.5496	0.1471	0.2758	0.8569
Indirect effect through cannabis use and cognitive biases	a_1_a_3_b_2_	0.3463	0.0704	0.2200	0.4958

Abbreviations: CI, confidence intervals; CT, childhood trauma; PLE, psychotic-like experiences; SE, standard error.

* *p* < 0.01.

** *p* < 0.001.

*** *p* < 0.0001.

There were significant direct effects of childhood trauma history, CPQ scores and DACOBS scores on the PQ scores. Similarly, the direct effects of childhood trauma history and CPQ scores on the DACOBS scores appeared to be significant in all models. Moreover, there were significant effects of childhood adversities on the CPQ scores. The total and indirect effects of childhood traumatic events on PQ-16 scores were also significant in all models. The percentage of variance in PQ-16 scores explained by serial mediation models varied between 32.8 and 34.2 (emotional neglect: *R* ^2^ = 0.3315, emotional abuse: *R* ^2^ = 0.3418, physical abuse: *R* ^2^ = 0.3370, sexual abuse: *R* ^2^ = 0.3279, any childhood adversities: *R* ^2^ = 0.3349).

The results of our study did not differ significantly when we performed analysis including individuals with a self-reported lifetime diagnosis of any mental disorder. The table with results will be included in Supplementary Materials (Table S1).

## Discussion

In our study, we found that the effect of childhood trauma on the level of PLEs is mediated by cannabis use and cognitive biases. The direct effect of childhood trauma history on the level of PLEs was also tested significant, suggesting that the mediation investigated in our study is partial. This finding is in line with results of previous studies [8,47]. When we analyzed different types of childhood trauma separately, the relationship with PLEs appeared to be most significant for emotional and physical abuse, which is in line with previous findings showing a different impact of specific traumatic events on the risk of psychosis [48]. Recent systematic review and meta-analysis on the association between childhood trauma with psychotic symptoms has shown that the early adversity is connected to severity of experienced symptomatology [49] There are also studies suggesting differential impact of particular types of childhood trauma and positive but not negative symptoms. Specifically, most consistently physical and sexual abuse has been associated with auditory hallucinations, but not delusions. Moreover, a number of studies have shown that childhood adversities are also related to the content of psychotic symptoms (for review see [50,51]).

We have shown the mediating role of cannabis use between trauma and PLEs. As presented by the recent systematic review of nonclinical populations, cannabis use is a risk factor for the development of PLEs [52]. It has been shown that similarly to the development of psychotic disorders [53], younger age at first use and higher frequency of cannabis use are associated with higher risk of PLEs in the general population [54,55]. Although evidence that cannabis use precedes the onset of psychotic symptoms and a dose–response relationship argue for a causal relationship, there are reports suggesting reversed causality. Indeed, experiencing psychotic symptoms may increase the risk for cannabis use. However, in the most recent population-based study on young adult twins, authors showed that there was a stronger support for a causal pathway from cannabis use to PLEs when compared to the opposite or reciprocal pathways after controlling for genetic and environmental factors [22]. Nevertheless, it should be noted that linear causality in psychiatry is unlikely and most psychic phenomena have complex causality with interdependent feedback loops.

Our results also indicate that cannabis use mediates the relationship between childhood and adolescent traumatic events and PLEs. This is in line with other studies that support our findings [13,27,37,38]. It has been shown that childhood adversities, in addition to cannabis use, may increase the risk of psychotic disorders [56,57] and that exposure to abuse and other life adversities together with cannabis use is associated with up to fourfold increased odds of reporting psychotic experiences [38]. Interestingly, a recent study showed that neither solely lifetime cannabis use nor reported exposure to childhood abuse was associated with increased risk of psychotic disorder, while the combination of the two risk factors substantially raised the likelihood of experiencing psychosis [58]. Moreover, exposure to childhood trauma and cannabis use were found to increase associations between hallucination and delusion in healthy and in genetically at risk populations thus increasing risk of transition to psychosis [59,60].

We found that early traumatic events as well as cannabis use are associated with cognitive biases that in turn contribute to the increased risk for psychotic experiences. It could be hypothesized that the additive interaction between early trauma and lifetime cannabis use produces alterations in salience processing, that is aberrant salience, and thus contributes to the development of cognitive biases that may in turn increase a risk of PLEs [30–32]. Two mechanisms of the emergence of aberrant salience have been proposed, both linked to stress and cannabis use. First, the role of cross-sensitization between stress and cannabis involving increased dopaminergic signaling in shaping the risk of psychotic outcomes has been suggested [61]. Second, it has been shown that both higher levels of cannabis use or childhood trauma compromise brain connectivity over the course of psychotic illness [62]. Both excessive dopamine signaling [63,64] and reduced cortico-striatal connectivity [65] have been associated with alterations in salience processing.

The aberrant salience model of psychosis proposes that chaotic brain dopamine transmission leads to the attribution of significance to stimuli that would normally be considered irrelevant [64]. Cognitive interpretation of these excessively salient stimuli can lead to the formation of biased cognitive schema that result in the formation of psychotic symptoms [66]. Hyperactivation in attentional systems and the biased schema, in turn, result in the excessively salient stimuli being interpreted as threatening and in the orientation of attention towards particularly threatening or anxiety-provoking environmental stimuli [67]. This process can give rise to attentional biases and as a result increase tendency for safety behaviors. For the review on the effect of aberrant signaling in dopaminergic neurotransmission on the development of cognitive biases see Broyd [67].

Aberrant salience has been shown to mediate the relationship between early trauma and PLEs [36,68]. It has been shown that individuals at ultra-high risk of psychosis demonstrate aberrant salience, the degree of which relates to the severity of delusion-like symptoms [69], while cannabis users show aberrant salience processing that is related to a severity of cannabis-induced psychotic symptoms [70]. Moreover, the positive relationship between aberrant salience and delusional symptoms has been described in schizophrenia patients [71].

Our study replicated the importance of cognitive biases for the emergence of PLEs. The role of cognitive biases in the relationship between early traumatic life events and PLEs has been reported previously [5,30,31,34]. The exposure to childhood adversities may cause deprivation of early social interactions, cognitive biases and disrupted attachment, which in turn may affect the development of neurocognition and social cognition observed in psychotic disorders [72].

Concluding, our results further support the hypothesis of childhood trauma having effect on psychosis proneness in the general nonclinical population. We have shown that most significant association can be observed between emotional and physical abuse and PLEs. Important mediators of the relationship between early trauma and PLEs are cannabis use and cognitive biases. Early traumatic events as well as cannabis use are associated with cognitive biases that in turn contribute to the increased risk for psychotic experiences. Although there are no studies showing the relationship between cannabis use and cognitive biases as assessed in our study, it can be speculated that trauma and cannabis use through increased dopamine synthesis and altered brain connectivity can promote aberrant salience that in turn may lead to higher occurrence of cognitive biases and PLEs.

## Limitations

There are some limitations of our study that need to be discussed. First, the questionnaires used in our study were shortened and the items were selected arbitrarily for the online screening purposes. Second, our study includes only self-report measures, that is measures that rely on the individual’s own report of their symptoms, beliefs, behaviors, or attitudes. Collecting information through self-report has its well-known limitations and disadvantages such as for example bias of how people feel at the time they fill out the questionnaire, recall bias and forgetting, or desirability bias. However, results from studies on trauma using self-report have been often validated by studies using other data methods that show that responses measure what they claim that they measure [51,73]. It has been shown that retrospective self-report can be used reliably to assess childhood trauma in people experiencing acute psychotic symptoms and that although the severity of childhood trauma reports can fluctuate between assessments, there are rarely complete retractions of severe abuse claims [73]. Moreover, given the cross-sectional nature of our study, we were unable to confirm causality between selected variables, thus limiting the clinical implications of our results. Prospective, longitudinal studies are required to examine the temporal course of trauma exposure, cannabis use, cognitive biases, and PLEs. Inclusion of biological measures that allow recording dopamine release or altered brain connectivity could provide further insights into our mediation model.

## Data Availability

The data that support the findings of this study are openly available on request to the all authors. Please contact corresponding author.

## Supplementary material

For supplementary material accompanying this paper visit http://dx.doi.org/10.1192/j.eurpsy.2020.31. click here to view supplementary material
