# Supplementary material for: The interplay between childhood trauma, cognitive biases, and cannabis use on the risk of psychosis in nonclinical young adults in Poland
Source: Eur Psychiatry. 2020 Mar 23;63(1):e35. doi: 10.1192/j.eurpsy.2020.31 (PMC7355126; doi:10.1192/j.eurpsy.2020.31)
Supplement: Supplementary file 1 [file S0924933820000310sup001.docx]

**Supplementary table 1**. Serial mediation analysis to identify direct and indirect effects of childhood trauma on psychotic-like experiences in the whole sample.

| Effect | Path | Coeff. | SE | 95%CI | |
| --- | --- | --- | --- | --- | --- |
|  |  |  |  | LLCI | ULCI |
| **Emotional abuse**  Direct effect of CT on cannabis use  Direct effect of CT on cognitive biases  Direct effect of cannabis use on cognitive biases  Direct effect of cannabis use on PLEs  Direct effect of cognitive biases on PLEs  Direct effect of CT on PLEs  Total indirect effect  Indirect effect through cannabis use  Indirect effect through cognitive biases  Indirect effect through cannabis use and cognitive biases | a_1_  a_2_  a_3_  b_1_  b_2_  c  ab  a_1_b_1_  a_2_b_2_  a_1_a_3_b_2_ | 0.9479*  2.4549*  1.2220*  0.7566*  0.2133*  1.6438*  1.4879  0.7171  0.5237  0.2471 | 0.1496  0.5144  0.1135  0.0747  0.0209  0.3221  0.2149  0.1410  0.1264  0.0553 | 0.6543  1.4454  0.9993  0.6100  0.1723  1.0115  1.0776  0.4546  0.2902  0.1490 | 1.2414  3.4645  1.4448  0.9031  0.2543  2.2760  1.9226  1.0079  0.7826  0.3630 |
| **Emotional neglect**  Direct effect of CT on cannabis use  Direct effect of CT on cognitive biases  Direct effect of cannabis use on cognitive biases  Direct effect of cannabis use on PLEs  Direct effect of cognitive biases on PLEs  Direct effect of CT on PLEs  Total indirect effect  Indirect effect through cannabis use  Indirect effect through cognitive biases  Indirect effect through cannabis use and cognitive biases | a_1_  a_2_  a_3_  b_1_  b_2_  c  ab  a_1_b_1_  a_2_b_2_  a_1_a_3_b_2_ | 1.0533*  2.5978*  1.3830*  0.7414*  0.2531*  1.5138*  1.8073  0.7810  0.6576  0.3687 | 0.1426  0.4669  0.0944  0.0711  0.0204  0.3227  0.2327  0.1400  0.1420  0.0716 | 0.7735  1.6816  1.1978  0.6019  0.2131  0.8718  1.3681  0.5248  0.3998  0.2440 | 1.3331  3.5114  1.5682  0.8810  0.2932  2.1559  2.2755  1.0739  0.9449  0.5183 |
| **Physical abuse**  Direct effect of CT on cannabis use  Direct effect of CT on cognitive biases  Direct effect of cannabis use on cognitive biases  Direct effect of cannabis use on PLEs  Direct effect of cognitive biases on PLEs  Direct effect of CT on PLEs  Total indirect effect  Indirect effect through cannabis use  Indirect effect through cognitive biases  Indirect effect through cannabis use and cognitive biases | a_1_  a_2_  a_3_  b_1_  b_2_  c  ab  a_1_b_1_  a_2_b_2_  a_1_a_3_b_2_ | 1.0117*  2.0724*  1.2354*  0.7567*  0.2181*  1.4951*  1.7338  0.7732  0.5871  0.3735 | 0.1508  0.5233  0.1142  0.0752  0.0209  0.3347  0.2227  0.1399  0.1348  0.0714 | 0.7158  1.0453  1.0112  0.6092  0.1771  0.8383  1.3190  0.5217  0.3413  0.2465 | 1.3076  3.0995  1.4596  0.9043  0.2591  2.1518  2.1844  1.0783  0.8633  0.5221 |
| **Sexual abuse**  Direct effect of CT on cannabis use  Direct effect of CT on cognitive biases  Direct effect of cannabis use on cognitive biases  Direct effect of cannabis use on PLEs  Direct effect of cognitive biases on PLEs  Direct effect of CT on PLEs  Total indirect effect  Indirect effect through cannabis use  Indirect effect through cognitive biases  Indirect effect through cannabis use and cognitive biases | a_1_  a_2_  a_3_  b_1_  b_2_  c  ab  a_1_b_1_  a_2_b_2_  a_1_a_3_b_2_ | 1.1046*  2.5705*  1.4177*  0.7506*  0.2572*  1.6629*  1.8931  0.8291  0.6612  0.4028 | 0.1761  0.5718  0.0942  0.0711  0.0204  0.3977  0.2939  0.1630  0.1770  0.0874 | 0.7592  1.4486  1.2329  0.6111  0.2173  0.8825  1.3334  0.5283  0.3258  0.2487 | 1.4500  3.6924  1.6026  0.8901  0.2972  2.4433  2.4902  1.1654  1.0285  0.5922 |
| **Any childhood adversities**  Direct effect of CT on cannabis use  Direct effect of CT on cognitive biases  Direct effect of cannabis use on cognitive biases  Direct effect of cannabis use on PLEs  Direct effect of cognitive biases on PLEs  Direct effect of CT on PLEs  Total indirect effect  Indirect effect through cannabis use  Indirect effect through cognitive biases  Indirect effect through cannabis use and cognitive biases | a_1_  a_2_  a_3_  b_1_  b_2_  c  ab  a_1_b_1_  a_2_b_2_  a_1_a_3_b_2_ | 1.3333*  2.2634*  1.4289*  0.7445*  0.2600*  1.7817*  2.0763  0.9926  0.5884  0.4935 | 0.1957  0.6417  0.0948  0.0714  0.0203  0.4436  0.2856  0.1738  0.1661  0.0925 | 0.9492  1.0044  1.2429  0.6045  0.2202  0.9113  1.5295  0.6688  0.2919  0.3296 | 1.7173  3.5224  1.6150  0.8845  0.2998  2.6521  2.6562  1.3431  0.9416  0.6947 |

*p < 0.001
